# Alcohol, tobacco and breast cancer: should alcohol be condemned and tobacco acquitted?

**DOI:** 10.1038/sj.bjc.6600633

**Published:** 2002-11-12

**Authors:** I dos Santos Silva

**Affiliations:** Department of Epidemiology and Population Health, London School of Hygiene and Tropical Medicine, Keppel Street, London WC1E 7HT, UK

## Abstract

*British Journal of Cancer* (2002) **87**, 1195–1196. doi:10.1038/sj.bjc.6600633 www.bjcancer.com

© 2002 Cancer Research UK

## 

The first published report of an association between alcohol consumption and breast cancer risk was by Williams and Horm (1977). This investigation was hypothesis-generating, however, in that it examined multiple potential risk factors for several cancers and, apart from age, ethnic group and smoking habits, no account was taken of other potential confounding variables. A possible association between breast cancer and tobacco smoking was first proposed by Macmahon *et al* (1982). They hypothesised that cigarette smoking would reduce the risk of breast cancer, mainly on the basis of their observation that smoking was associated with a reduction in urinary oestrogen levels during the luteal phase of the menstrual cycle. However, Hiatt and Fireman (1986) proposed a contrary hypothesis. They postulated that tobacco smoke would have a direct carcinogenic effect as mutagens from cigarette smoke had been found in the breast fluid of non-lactating women.

Since these initial reports, a substantial number of epidemiological investigations have assessed these hypotheses. The overall evidence from the epidemiological data published so far seems to indicate that alcohol intake may be associated with a slight increase in the risk of breast cancer. By contrast, the evidence for smoking has been rather inconsistent, with some studies showing a slight increase in risk and others reporting no association or, even, a protective effect. Most of these studies have been based on relatively small numbers of breast cancer cases, however, thus yielding rather imprecise estimates of the true effects of these exposures. In addition many of them were unable to take account of the fact that alcohol and tobacco consumption are correlated, as smokers are more likely to drink alcohol than non-smokers.

Clarification of the independent effects, if any, of these exposures on the risk of breast cancer is of epidemiological relevance for various reasons. First, for populations where the prevalence of alcohol and/or tobacco consumption is high, even if the relative risk associated with these exposures is small, the effect of these exposures could still account for a substantial number of breast cancer cases. Second, unlike most of the known risk factors for breast cancer, alcohol and tobacco consumption are potentially modifiable behaviours and hence offer some scope for prevention. Third, clarification of the aetiological role of these exposures might shed light on the biology of breast cancer.

In this issue, the Collaborative Group on Hormonal Factors in Breast Cancer (2002) has published a reanalysis of individual data from over 80% of the worldwide epidemiological information on alcohol, tobacco and breast cancer risk in women. This reanalysis of data from 53 different studies, which included 58 515 women with breast cancer and 95 067 without, showed that the relative risk of breast cancer increased by 7.1% for each additional 10 g day^−1^ intake of alcohol, i.e. for each extra unit (alcoholic drink) consumed on a daily basis. Relative to women who reported drinking no alcohol, the risk of developing breast cancer was 32% higher among those who reported drinking 35–44 g day^−1^ and 46% higher among those who reported consuming 45 g day^−1^ or more (average 57 g day^−1^). The effect of alcohol was not found to be confounded by smoking or by any other known risk factor for breast cancer. The authors estimated that in developed countries the cumulative incidence of breast cancer by age 80 would be 8.8 per 100 women in non-drinkers and 9.4, 10.1, 10.8, 11.6, 12.4 and 13.3, respectively, per 100 women among women consuming an average of 1, 2, 3, 4, 5 and 6 alcoholic drinks each day. By contrast, the relationship between smoking and breast cancer was substantially confounded by the effect of alcohol and when the analysis was restricted to non-drinkers, no association was found between smoking and breast cancer.

So, can we now finally pass judgement on the alcohol- and tobacco-breast cancer hypotheses and lay them to rest? Unfortunately, the answer is not yet. As acknowledged by the authors the quality and validity of their exposure data were, to a certain extent, limited, as most of the individual studies were not set up with the primary purpose of addressing these hypotheses. Despite this caveat, the results strongly argue against an association between smoking and breast cancer but are in favour of a true positive association between alcohol and this tumour. Given the impossibility of conducting a randomised trial on this issue a causal relation between alcohol and breast cancer can never be established beyond any doubt, but the evidence provided by this reanalysis of practically all the available worldwide data, and the consistency of the findings across the various studies, strongly suggest that something is going on. As the relative risks involved are modest, it could be argued that the observed relationship between alcohol and breast cancer might be due to confounding by unknown risk factors, but it is hard to envisage an as yet unknown risk factor, which is consistently associated with alcohol intake in the relatively diverse populations studied.

Even if causal, the exact relationship with duration, timing, or indeed amount, of alcohol intake remains unclear. Self-reported information on alcohol consumption is known to be prone to measurement error and it is uncertain to what extent this might have affected the magnitude and shape of the dose–response relationship. Thus, a degree of uncertainty remains about the true quantitative effect of a given amount of alcohol on the risk of developing breast cancer, and in particular, whether there may be a threshold dose below which alcohol has no effect. The tendency for underestimation of the amount drunk and concerns that non-drinkers may differ in some relevant, but unmeasured, ways from those who occasionally drink alcohol, precludes an accurate estimation of the risks associated with low levels of alcohol intake.

Should the advice to women change in the light of the findings from this reanalysis? After assimilating the new findings the answer is probably no. In terms of public health, both factors are already known to be important for women's health. Smoking has already been shown to cause many fatal diseases, particularly lung cancer and coronary heart disease. The implications of the findings for alcohol are less straightforward. In this reanalysis, the average consumption of alcohol reported by controls from developed countries was 6.0 g day^−1^. On the basis of the observed dose–response relationship, and assuming causality, the authors estimated that about 4% of all breast cancer cases in developed countries are attributable to alcohol. In developing countries, where the average alcohol consumption is very low (only 0.4 g day^−1^), alcohol would make a negligible contribution to the total number of cases of breast cancer. To put these figures into perspective, up to 90% of all lung and cervical cancers can be attributed to smoking and infection with human papillomavirus (HPV), respectively.

Complete elimination of alcohol consumption is neither feasible nor justified in health terms. We know from other studies that women who drink alcoholic beverages regularly have higher death rates from injuries, violence, suicide, poisoning, cirrhosis, certain cancers (namely cancers of the oropharynx, larynx, oesophagus and liver), and possibly haemorrhagic stroke, but lower death rates from coronary heart disease and thrombotic stroke compared with those who abstain completely (Thun *et al*, 1997). The net balance of risks and benefits associated with alcohol intake is, therefore, complex. The American Cancer Society (ACS) study of half a million people who have been followed since 1982, suggests that all-cause mortality rates are 20% lower among middle aged women who drink one alcoholic drink per day relative to those who abstain, and only begin to show an increase at around two drinks per day (Thun *et al*, 1997). In a recent evaluation of alcohol-related mortality in England and Wales (Britton and Mcpherson, 2001), a net protective mortality balance could only be found among women aged over 65 years, the age after which the risk of coronary heart disease is highest. By contrast, breast cancer was one of the top three causes of alcohol related deaths in younger women together with alcoholic liver diseases and suicide. It has been estimated that the most beneficial drinking levels in terms of lowest risk of death for women in England and Wales would be zero consumption at ages 16 to 54 years, rising to 3 units per week in those aged over 65 years (White *et al*, 2002), but these estimates do not allow for the fact that many alcohol-related effects, including the cardioprotective ones, may require exposure over several years before becoming apparent.

The answer to the initial question ‘Should alcohol be condemned and tobacco acquitted?’ will depend on whether this is primarily an aetiological or a public health question. In terms of breast cancer aetiology, this reanalysis provides strong evidence that alcohol, rather than smoking, is the culprit. And yet, in public health terms, there is no doubt that tobacco should be condemned as it was responsible for an average of about 3 million deaths per year worldwide during the 1990s and, if nothing is done to change current smoking patterns, this figure may well rise to 10 million deaths per year by the 2020s (Peto *et al*, 1994). Alcohol intake, on the other hand, is likely to account, at present, for a small proportion of breast cancer cases in developed countries, but for women who drink moderately, its lifetime cardioprotective effects probably outweigh its health hazards.
